# Traditional Chinese medicine on treating pain caused by prostate cancer: A systematic review and meta-analysis

**DOI:** 10.1097/MD.0000000000017624

**Published:** 2019-11-01

**Authors:** Ning Wang, Li Xu, Ji-Sheng Wang, Xu-Dong Yu, Li-Yuan Chu, Sheng Deng, Fu-Xing Ge, Hai-Song Li

**Affiliations:** a Ningxia Zhongwei City Chinese Medicine Hospital; b Graduate School of Beijing University of Chinese Medicine; c Department of Andrology; d Department of Oncology, Dongzhimen Hospital; e School of Chinese Material, Beijing University of Chinese Medicine, Beijing, China.

**Keywords:** pain, prostate cancer, protocol, systematic review, traditional Chinese medicine

## Abstract

**Introduction:**

Prostate cancer is a male malignant tumor disease with high prevalence in recent years. Patients with advanced prostate cancer are more likely to have bone metastasis and have strong bone pain, and even lead to pathological fracture, which has a serious impact on the quality of life of patients. Traditional Chinese medicine has good clinical efficacy in treating pain caused by prostate cancer .This review hopes to adopt meta-analysis to evaluate the efficacy and safety of TCM in the treatment of pain caused by prostate cancer and provide evidence for its application in clinical practice.

**Methods and analysis:**

We will search for PubMed, Cochrane Library, AMED, EMbase, WorldSciNet; Nature, Science online and China Journal Full-text Database (CNKI), China Biomedical Literature CD-ROM Database (CBM), and related randomized controlled trials included in the China Resources Database. The time is limited from the construction of the library to June 2019.We will use the criteria provided by Cochrane 5.1.0 for quality assessment and risk assessment of the included studies, and use the Revman 5.3 and Stata13.0 software for meta-analysis of the effectiveness, recurrence rate, and symptom scores of pain caused by prostate cancer.

**Ethics and dissemination:**

This systematic review will evaluate the efficacy and safety of TCM for pain caused by prostate cancer. Because all of the data used in this systematic review and meta-analysis has been published, this review does not require ethical approval. Furthermore, all data will be analyzed anonymously during the review process Trial.

**Trial registration number:**

PROSPERO CRD42019131544

## 1 Introduction

Prostate cancer is a male malignant tumor disease which has a high prevalence in recent years.^[1]^ Patients who suffer from advanced prostate cancer are vulnerable to osseous metastasis which will lead to intense pain, so far as to pathological fracture, which has tremendous reduction in the quality of life.^[2]^ The secretion of prostaglandin could accelerate the bone resorption around the tumor. Due to the intense sensibility of the nerve endings, sharp pain hence generate.^[3]^ According to vast research, it has proved that traditional Chinese medicine could be efficient in analgesic therapy.^[4,5]^ Recent years have witnessed great promotion of the living standard, which also accompanied with the increasing morbidity of prostate cancer, and this has caused great impact on people's life and quality of life. Meanwhile, with the increasing progress of modern medicine, we could not only focus on prolonging lifespan of the cancerous person, but also on the impact to the quality of life.^[6,7]^ Enormous clinical literatures have indicated that TCM has positive effect on ameliorating pain and improve the quality of life in patients who suffer from advanced prostate cancer.^[8,9]^

Prostate cancer is the most common malignant tumor of the male genitourinary system, accounting for the fifth place in the global cancer rate. According to the WHO Global Cancer Epidemiology Statistics (GLOBOCAN 2008), the global morbidity of prostate cancer ranks second in males who suffer from malignancy in 2008 (ranking only second to lung cancer), which accounting for 14%.^[10]^ Meanwhile, the morbidity also has significant difference across the world, which we found that the highest incidence, comparing with the lowest incidence, is about 25 times. In the United States, the morbidity of the disease ranks first in all male malignancies, which accounting for about 29%. During 2004 to 2008, the morbidity of this cancer had been turned to 152.9 per 100,000, which was counted after the rise of age.^[11,12]^

Cancer pain is one of the most important symptoms in patients who suffer from advanced cancer, and it greatly deteriorate the physical and psychological health, meanwhile, the quality of life of patients.^[13,14]^ The morbidity of the symptom is about 70% to 90%. According to the statistics which announced by WHO in 2003, there are about 10 million new cancer patients in the world every year, and 300 to 1000 million cancer patients fail to receive timely and effective treatment.

At present, aiming at the symptom, the medical community mainly adopts the “3-step” analgesic drug therapy recommended by WHO, which means that the patients need long-term high-dose anesthetics in order to release the pain.^[15]^ Meanwhile, studies in the United States have found that using large doses of anesthetics for a long period of time is vulnerable to promoting tumor angiogenesis, accelerating tumor growth and increasing the spread of cancer cells.^[16]^ Besides, patients who accept the treatment always accompanied with constipation, nausea and vomiting, dizziness, drowsiness, rash, high blood pressure, coma and other adverse reactions, so far as to further aggravating the pain.^[17,18]^

TCM has been widely used in clinical trials of pain caused by prostate cancer in recent years.^[8]^ It can relieve pain caused by prostate cancer to some extent.^[9]^ On the basis of TCM theory, Chinese herbal compound can regulate the balance of qi and blood, which aims at improving physiological function. Meanwhile, vast studies have shown that some ingredients in traditional Chinese medicine can adjust the production of neurotransmitter, mainly 5-HT, and reduce nerve sensitivity.^[19]^ By preliminary database searching, we found that the randomized controlled trials about TCM for curing pain caused by prostate cancer have been increasing. However, most clinical trials confront with the inferior quality of the studies with small sample size and the insufficiency of evidence-based exploration because of the limitation of the size and number of clinical centers. Therefore, we expect to adopt meta-analysis to evaluate the efficacy and safety of TCM in the treatment of prostate cancer, which in order to provide evidence for its clinical application.

TCM has its unique advantages in the treatment of cancer pain. The operation is convenient, it could dredge the meridians, regulate the balance of qi and blood, which achieve the goal of relieving pain caused by qi stagnation and blood stasis and meridians impassability.^[20]^ The process of analgesia by TCM is complicated. Vast studies have proved that Chinese herb extracts can accelerate the secretion of various mediators and opioid peptides from peripheral nerve to central nervous system, such as spinal cord, low brainstem, diencephalon, limbic system and cerebral cortex, which together form the “anti-pain system” of the human body and produce

Chinese herbal medicine analgesic effects.^[21]^ With the deepening of the research and animal experiment on the mechanism of TCM and analgesia in recent years, there have been increasing reports on the application of TCM to various acute and chronic pain treatments.

## 2 Methods

This systematic review protocol has been registered on PROSPERO as CRD42019131544. The protocol follows the Cochrane Handbook for Systematic Reviews of Interventions and the Preferred Reporting Items for Systematic Reviews and Meta-Analysis Protocol (PRISMA-P) statement guidelines. We will describe the changes in our full review if needed.

### 2.1 Inclusion criteria for study selection

#### 2.1.1 Types of studies

The selected literature will include the TCM-related randomized controlled trials which aim at treating pain caused by prostate cancer. The language is limited to Chinese and English. Non-randomized controlled trials, quasi-randomized controlled trials, case series, case reports, crossover studies will be excluded.

#### 2.1.2 Types of participants

Male patients who were diagnosed with prostate cancer will be included, the type of the disease includes adenocarcinoma (adenocarcinoma), ductal adenocarcinoma, urothelial carcinoma, squamous cell carcinoma, and adenosquamous carcinoma. Meanwhile, the patients who were recruited should have the symptom of pain caused by prostate cancer, including bone pain caused by bone metastasis. However, the patients who suffer from pain caused by other factors would be excluded. Patients who suffer from cognition impairment and other severe mental illness will also be excluded. In addition, the inclusion criteria will not be restricted by region, country, nation, and origin.

#### 2.1.3 Types of interventions

##### 2.1.3.1 Experimental interventions

The drug composition, the dose-specific Chinese medicine preparation or the combined western medicine are used as experimental interventions. Both prescription and Chinese patent medicines will be included. Other traditional Chinese medicine treatments such as intravenous medication, acupuncture, and massage will be limited.

##### 2.1.3.2 Control interventions

As for the control interventions, who accepted simple western medicine can be used as a control intervention or didn‘t get any treatment as a blank control would be adopted. However, once they had accepted the therapy of TCM, the trials will be rejected.

#### 2.1.4 Types of outcome measures

##### 2.1.4.1 Primary outcomes

Adopting NRS (Numerical rating scale) of pain criteria as the main evaluation, the specific division: Painless 0 points; mild pain 1 to 3 points; moderate pain 4 to 6 points; severe pain 7 to 9 points; severe pain 10 points, 0 to 10 points representing the patient's pain level. Significantly effective: the patient's pain level is reduced by at least 2 or 3 levels, or the patient is painless; Effective: The patient's pain level is reduced by one grade or the patient presents moderate and mild pain; Invalid: The patient's pain does not have any relief, and there is even a tendency to aggravate.

##### 2.1.4.2 Secondary outcomes

(1) International prostate symptom score (IPSS), the patient self-evaluation form;
(2) Quality of life scores for patients with prostate cancer before and after treatment (PRSSQOL): A scale, designed by the University of California, USA, to evaluate the condition and living quality of patients who suffer from advanced, hormone-insensitive prostate cancer. It specifically includes nine aspects: Physical strength, pain, fatigue, appetite, family/marriage, constipation, mood, defecation, and overall feelings. Each item is calculated with 100 points, and the lower the score, the worse the situation.

### 2.2 Search methods for the identification of studies

#### 2.2.1 Electronic searches

Database search: Searching PubMed, Cochrane Library, AMED, EMbase, WorldSciNet; Nature, Science online and China National Knowledge Infrastructure (CNKI), China Biology Medicine disc (CBM), China Resources Database. The temporal interval is limited from the time that the databases created to June 2019, and the combination of keyword and free word retrieval is adopted. The search terms include “Chinese medicine”, “traditional Chinese medicine”, “proprietary Chinese medicine”, “Chinese herbal medicine”, “prostate cancer”, “prostate cancer”, “pain”. “Random Control”. The complete PubMed search strategy is summarized in Table 1.

**Table 1:**
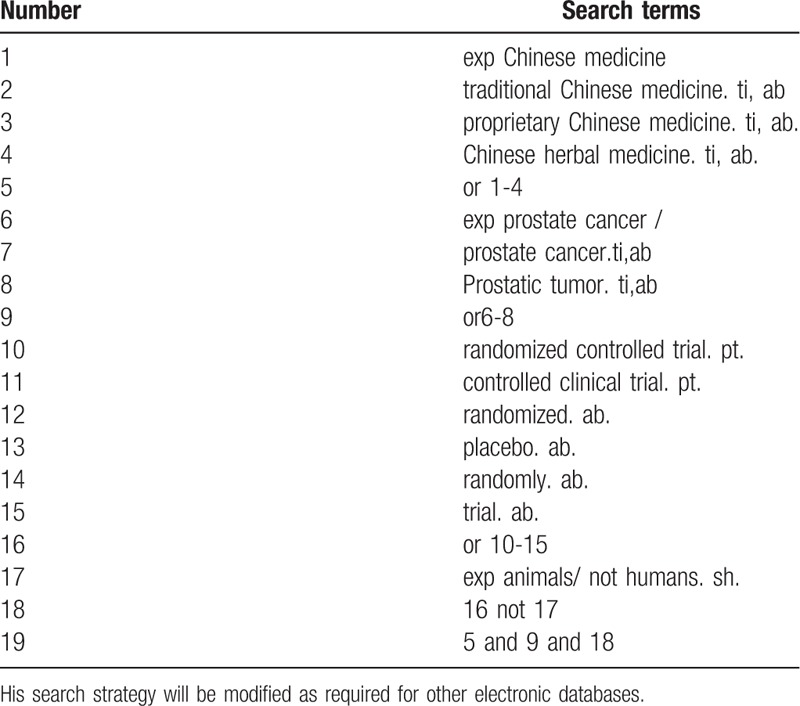
Search strategy used in PubMed database.

#### 2.2.2 Searching other resources

We will search ongoing experimental studies related to the disease through the WHO International Clinical Trial Registration Platform (ICTRP), ClinicalTrials.gov, and Chinese clinical trial studies. As for these ongoing experiments, we will try to contact the trial author to help provide the most up-to-date clinical data. Besides, we also tempt to adopt the manual searching which mainly aims at relevant literatures, earlier than the database above-mentioned, such as “China Rehabilitation Medicine Journal” “Chinese Journal of Physical Medicine and Rehabilitation” and “Chinese Journal of Urology”.

### 2.3 Data collection and analysis

#### 2.3.1 Selection of studies

Initial evaluations were performed independently by 2 investigators through filtrating the titles and abstracts of each documents in the Endnote database, eliminating duplicates and documents that were clearly inconsistent with the study. After the preliminary assessment, in order to screen out eligible trials, the full text of the selected literature would be evaluated, which mainly aims at whether there were problems just like uncontrolled studies, no randomization, inconsistent assessment criteria, and similar data. When the two researchers could not reach a consensus, the third judge would make the final judgment.

#### 2.3.2 Data extraction and management

There will be 2 investigators independently extract information from the included literature, mainly contents: Author (year), sample size, disease stage, patient age, intervention factors, control factors, intervention time, observation index, NRS score, symptoms Ratings, quality of life scores, etc. The extracted literature data is filled in a unified data statistics table.

#### 2.3.3 Assessment of risk of bias in included studies

Two investigators independently assess the quality of the included literature by using the Cochrane Collaboration's bias risk assessment tool. The assessment includes: random sequence generation, allocation concealment, blinding, incomplete outcome data, selective outcome reporting, and other possible biases. According to the relevant standards in the *Cochrane Intervention System Evaluation Manual*, it is divided into low risk, high risk and unclear.

#### 2.3.4 Dealing with missing data

In the event of data loss during the screening and extraction of literature data, primarily, we would actively look for the cause of the loss, and then we would contact the experimental research author by telephone, mail, etc. to retrieve the lost data. If the loss could not be retrieved, we will only extract and analyze the useful data, and the situation would be indicated.

#### 2.3.5 Data synthesis and analysis

**Figure 1:**
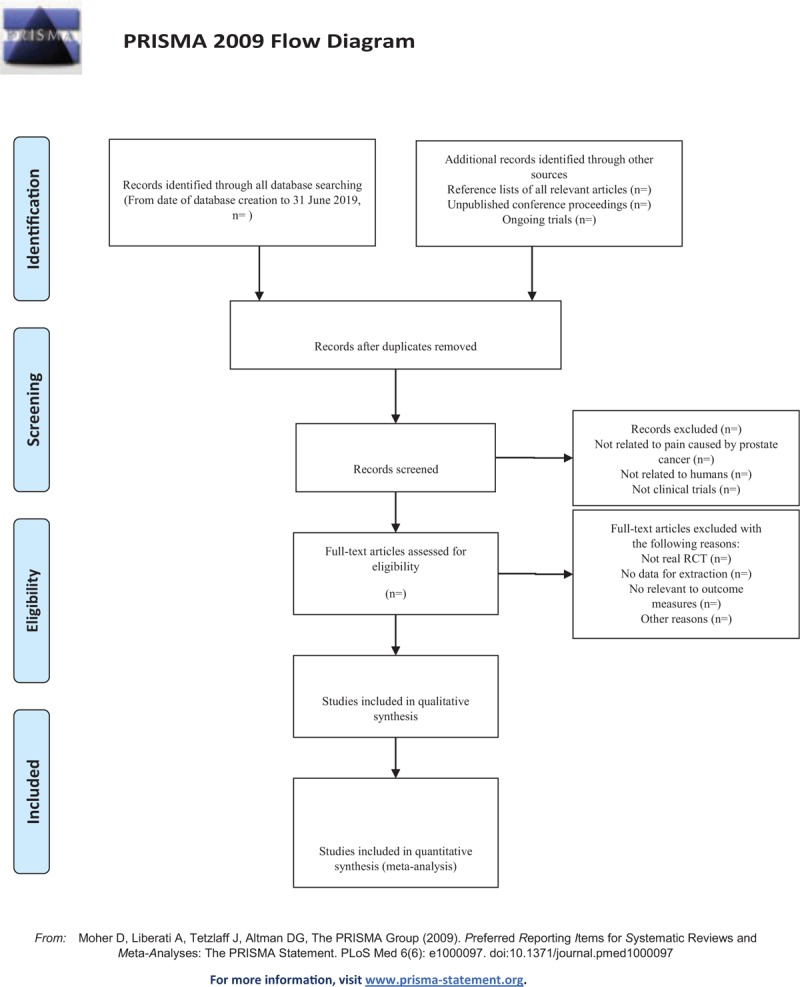
The PRISMA flow chart.

The analysis of the data will adopt RevMan 5.3 software. As for the two categorical variables, we select relative risk (RR) or odds ratio (OR) and 95% CI. As for the continuous variables, we select weighted mean difference (WMD) or standard mean difference (SMD) and 95% CI, the difference would be statistically significant when *P* < .05. Heterogeneity test would be analyzed by using chi-square test. When *P* ≥ .1, the difference was considered to be not statistically significant. When *P* < .1, I^2^ > 50%, the random effect model would be used, as for the other situation, the fixed effect model would be adopted. For studies that provide baseline and post-treatment data, we will estimate the change values by the method recommended by Cochrane. The details of selection process will be shown in the PRISMA flow chart (Fig. 1).

#### 2.3.6 Assessment of heterogeneity

If there is significant heterogeneity between a group of studies, we will explore the reasons for the existence of heterogeneity from various aspects, such as the characteristics of the subjects and the degree of variation of the interventions. Necessarily, sensitivity analysis or subgroup analysis would be adopted to explain the heterogeneity.

#### 2.3.7 Assessment of publication bias

The forest map and funnel plot would be drawn and analyzed by Rev Man5.3 software, and the funnel plot would be used to analyze the potential publication bias.

#### 2.3.8 Grading the quality of evidence

The quality of evidence for the main outcomes will also be assessed with the GRADE approach. The evaluation included bias risk; heterogeneity; indirectness; imprecision; publication bias. And each level of evidence will be made “very low,” “low,” rate,” or “high” judgment.

**Patient and public involvement**

No patients will be involved in this study.

## 3 Discussion

At present, pain has been the fifth vital sign, which together with respiration, blood pressure, pulse and body temperature. In 1996, the American Pain Association (APA) first proposed the concept of pain and its control, which indicated that an effective pain relief program for patients is a basic requirement for clinical medical work. It has been a difficulty problem to the treatment of pain caused by prostate cancer and its bone metastasis in the middle and advantaged stage, which has belonged to refractory cancer pain.^[22]^ Recent years have witnessed an increase on the study of treating cancer pain, vast researches have indicated that analgesics have great efficiency in ameliorating pain, but the pesticide effects are short, and there are kinds of adverse reaction, such as kidney damage, liver damage, cardiovascular and cerebrovascular damage, digestive disease, etc.^[23,24]^ Generally speaking, numerous patients who suffer from cancer could not gain satisfied control effect. Moreover, these patients need long-term use of analgesic drugs to ameliorate pain, which also directly increase the cost of therapy for patients.

Traditional Chinese medicine, possessing thousands of years history in China, has been proved that it is a safe, feasible and effective treatment. The clinical operation and theory of TCM have been inherited and carried forward by the majority of clinicians, especially in the treatment of pain. This therapy could ameliorate vast kinds of pain efficiently, and there are not adverse reaction which could insure the security.^[25]^ In recent years, TCM therapy has been widely used in clinical trials of pain caused by prostate, and the recent studies have shown that the therapy could ameliorate the pain cause by prostate cancer to some extent and improve the quality of the life.^[26]^

As far as we know, there has not made any comparison of the effectiveness of TCM in the treatment of prostate cancer pain. Therefore, we will use systematic review and meta-analysis to evaluate the efficacy and safety of TCM for the treatment of pain caused by prostate cancer. We expect that the review could provide a basis for TCM treatment of pain caused by prostate cancer and offer more and better options for the treatment to patients. In addition, the literature on TCM therapy on pain caused by prostate cancer is relatively inadequate and the overall quality is a little low, which may affect the authenticity of this study.

**Ethics and dissemination**

This systematic review will evaluate the efficacy and safety of TCM for pain caused by prostate cancer. Because all of the data used in this systematic review and meta-analysis has been published, this review does not require ethical approval. Furthermore, all data will be analyzed anonymously during the review process Trial.

## Author contributions

**Conceptualization:** Ning Wang.

**Data curation:** Ning Wang.

**Investigation:** Li Xu, Ji-Sheng Wang.

**Methodology:** Ji-Sheng Wang.

**Project administration:** Ji-Sheng Wang, Sheng Deng, Fu-Xing Ge.

**Resources:** Xu-Dong Yu, Li-Yuan Chu, Sheng Deng, Fu-Xing Ge.

**Software:** Xu-Dong Yu.

**Validation:** Li-Yuan Chu.
